# Population-scale Ancestral Recombination Graphs with tskit 1.0

**Published:** 2026-09-08

**Authors:** Ben Jeffery, Yan Wong, Kevin Thornton, Georgia Tsambos, Gertjan Bisschop, Yun Deng, E. Castedo Ellerman, Thomas B. Forest, Halley Fritze, Daniel Goldstein, Gregor Gorjanc, Graham Gower, Simon Gravel, Jeremy Guez, Benjamin C. Haller, Andrew D. Kern, Lloyd Kirk, Ivan Krukov, Hanbin Lee, Brieuc Lehmann, Hossameldin Loay, Matthew M. Osmond, Duncan S. Palmer, Nathaniel S. Pope, Aaron P. Ragsdale, Duncan Robertson, Murillo F. Rodrigues, Hugo van Kemenade, Clemens L. Weiß, Anthony Wilder Wohns, Shing H. Zhan, Brian C. Zhang, Marianne Aspbury, Nikolas A. Baya, Saurabh Belsare, Arjun Biddanda, Francisco Campuzano Jiménez, Ariella Gladstein, Bing Guo, Savita Karthikeyan, Warren W. Kretzschmar, Inés Rebollo, Kumar Saunack, Ruhollah Shemirani, Alexis Simon, Chris Smith, Jeet Sukumaran, Jonathan Terhorst, Per Unneberg, Ao Zhang, Peter Ralph, Jerome Kelleher

**Affiliations:** 1 Big Data Institute, Li Ka Shing Centre for Health Information and Discovery, University of Oxford, OX3 7LF, UK; 2 Department of Systems Biology, University of California, Irvine, CA 92697, USA; 3 Melbourne Integrative Genomics, School of Mathematics and Statistics, University of Melbourne, Victoria, 3010, Australia; 4 Department of Genome Sciences, University of Washington, Seattle, WA 98195, USA; 5 Department of Genetics, Stanford University, Stanford, CA 94305, USA; 6 Fresh Pond Research Institute, Cambridge, MA 02140, USA; 7 Institute of Ecology and Evolution, University of Oregon, Eugene OR 97402, USA; 8 Department of Statistics, University of Michigan, Ann Arbor, MI, 48109, USA; 9 Khoury College of Computer Sciences, Northeastern University, MA 02115, USA; 10 The Roslin Institute and Royal (Dick) School of Veterinary Studies, University of Edinburgh, EH25 9RG, UK; 11 Microbiology and Infectious Diseases, SA Pathology, Adelaide, SA 5000, Australia; 12 Department of Human Genetics, McGill University, Montreal, QC H3A 0C7, Canada; 13 UMR 7206 Eco-Anthropologie, CNRS, MNHN, Université Paris Cité, 75116 Paris, France; 14 Université Paris-Saclay, CNRS, INRIA, Laboratoire Interdisciplinaire des Sciences du Numérique, 91400, Orsay, France; 15 Dept. of Computational Biology, Cornell University, Ithaca, NY 14853, USA; 16 Department of Integrative Biology, University of Wisconsin–Madison, WI 53706, USA; 17 Department of Statistical Science, University College London, London, WC1E 7HB, UK; 18 Department of Ecology and Evolutionary Biology, University of Toronto, Toronto, Ontario, M5S 3B2, Canada; 19 Department of Statistics, University of Oxford, OX1 3LB, UK; 20 The Pioneer Centre for SMARTbiomed, Big Data Institute, Li Ka Shing Centre for Health Information and Discovery, University of Oxford, OX3 7LF, UK; 21 Program in Medical and Population Genetics, Broad Institute of MIT and Harvard, Cambridge, Massachusetts 02142, USA; 22 Independent researcher; 23 Department of Genetics, Stanford University School of Medicine, Stanford, CA 94305, USA; 24 Stanford Cancer Institute, Stanford School of Medicine, Stanford, CA 94305, USA; 25 Infectious Disease Epidemiology Unit (IDEU), Nuffield Department of Population Health, University of Oxford, OX3 7LF, UK; 26 Department of Paediatrics, University of Oxford, Oxford, UK; 27 Department of Biology, Johns Hopkins University, Baltimore, MD, 21218, USA; 28 Department of Biology, University of Antwerp, Antwerp, 2610, Belgium; 29 Department of Human Genetics, University of California, Los Angeles, CA 90095, USA; 30 Center for Vaccine Development and Global Health, University of Maryland School of Medicine, Baltimore, MD, 21201, USA; 31 Institute for Genome Sciences, University of Maryland School of Medicine, Baltimore, MD, 21201, USA; 32 Center for Hematology and Regenerative Medicine, Karolinska Institute, 141 83 Huddinge, Sweden; 33 Instituto Nacional de Investigacíon Agropecuaria (INIA), Estacíon Experimental Las Brujas, Ruta 48 km 10, Canelones, Uruguay; 34 Department of Statistics, Universidad de la República, College of Agriculture, Garzón 780, Montevideo, Uruguay; 35 Institute for Genomic Health, Icahn School of Medicine at Mount Sinai, NY, 10029, USA; 36 Sorbonne Université, CNRS, UMR 7144 AD2M, DiSEEM, Station Biologique de Roscoff, France; 37 Biology Department, San Diego State University, San Diego, CA 92182-4614, USA; 38 Department of Cell and Molecular Biology, National Bioinformatics Infrastructure Sweden, Science for Life Laboratory, Uppsala University, Husargatan 3, SE-752 37 Uppsala, Sweden; 39 Department of Data Science, University of Oregon, Eugene OR 97402, USA

Ancestral recombination graphs (ARGs) capture the full genetic history of samples from a recombining species. Although ARGs have been a central theoretical object in population genetics for decades, their practical use was constrained by the lack of scalable inference methods, standard interchange formats, and software infrastructure. Recent breakthroughs in simulation and inference have substantially changed this landscape, leading to renewed interest in ARG-based analyses across population and statistical genetics (Brandt et al., 2024; Lewanski et al., 2024; Nielsen et al., 2024). The tskit library has played a key enabling role in this shift and has become foundational infrastructure for working with ARGs. This paper marks the release of tskit 1.0, which formalises long-term stability guarantees for its data formats and APIs.

At the core of tskit is the succinct tree sequence data model which defines a set of nodes (genomes at particular times) and edges (inheritance relationships between nodes spanning genomic intervals) in a simple tabular form (Kelleher et al., 2018). This encoding provides a lossless representation of a general class of ARGs, establishing a precise and machine-readable definition suitable for large-scale computation (Wong et al., 2024). The data model also incorporates site, mutation, population, and pedigree information and supports arbitrary metadata associated with each of these components of the ARG. Provenance information is recorded natively, enhancing reproducibility and transparency. Together, these features make the tskit data model a semantically complete and interoperable representation of ARGs that serves as a common foundation across diverse analytical workflows (Figure 1).

Simulation is a fundamental tool in population genomics, and was the first domain in which the tskit data model demonstrated its impact. Introduced initially as part of the msprime simulator, the tskit data model enabled performance improvements of several orders of magnitude over previous coalescent simulation approaches (Kelleher et al., 2016). The same representation later enabled efficient forward-time simulation of ARGs and yielded substantial speedups by avoiding explicit simulation of neutral mutations (Kelleher et al., 2018). Because these forward-time and coalescent simulators share a common underlying representation, their complementary strengths can be combined within a single workflow. This has made it possible to simulate ARGs under complex demographic scenarios involving geography and selection that were previously infeasible, providing essential ground truth for method evaluation. Simulation capabilities have continued to expand, including whole-autosome ARG simulations for nearly 1.5 million individuals based on a large human pedigree (Anderson-Trocmé et al., 2023). A growing ecosystem of forward-time, coalescent, and hybrid simulation tools builds directly on tskit (Table S1).

The lack of scalable inference methods has been a major obstacle to practical application of ARGs. Although there are many inference methods (see Wong et al. (2024) for a review), tsinfer was the first to scale to hundreds of thousands of samples, directly leveraging the tskit data model (Kelleher et al., 2019). Many recent ARG inference methods have chosen to support tskit as an output format in addition to their own native representations (Table S1). This shared output layer enables inferred ARGs to interoperate directly with simulators, facilitating systematic evaluation and benchmarking against known ground truth. It also shifts the burden of format conversion away from downstream users, who can instead rely on inference tools to emit results in a common, well-defined representation. The scalability and flexibility of this approach are illustrated by the recent inference of an ARG for 2.48 million SARS-CoV-2 whole genomes, which occupies 32 MiB of storage and can be loaded into memory in under a second (Zhan et al., 2025).

Efficient storage and analysis of large genetic datasets is a central design goal of tskit, and the data model has enabled substantial performance gains in downstream analyses. For example, single-site population genetic statistics can be computed orders of magnitude faster than from genotype matrices while using far less memory by operating on the underlying ARG structure (Ralph et al., 2020). Tskit exposes a large API with a performance-critical core implemented in C and bindings available for Python, Rust, and R. Its vectorised, table-first design allows zero-copy access to underlying arrays, supporting high-performance analysis pipelines. As a result, downstream tools inherit performance and correctness properties from a shared, well-tested core.

The goal of tskit is to provide a shared technical foundation, centred on efficient, well-tested, and thoroughly documented primitive operations on ARGs, rather than to directly implement end-user workflows. This design principle has enabled a broad ecosystem of downstream software—spanning simulation, ARG inference, population and statistical genetic inference, analysis, and visualisation—with 64 published tools now using tskit as a core dependency (Table S1). With the release of tskit 1.0, long-term stability of the data model and APIs is explicitly guaranteed, reflecting a commitment to maintain this minimal and composable core as the project evolves. By focusing on stable primitives rather than prescribing analytical pipelines, tskit enables methodological innovation to concentrate on modelling, inference, and interpretation rather than bespoke data formats and tooling. In this way, tskit 1.0 provides a common and extensible foundation that supports the continued expansion of ARG-based analyses as datasets, methods, and applications continue to grow. Extensive documentation, tutorials, and other information are available at https://tskit.dev.

## Supplementary Material

1

## Figures and Tables

**Figure 1:**
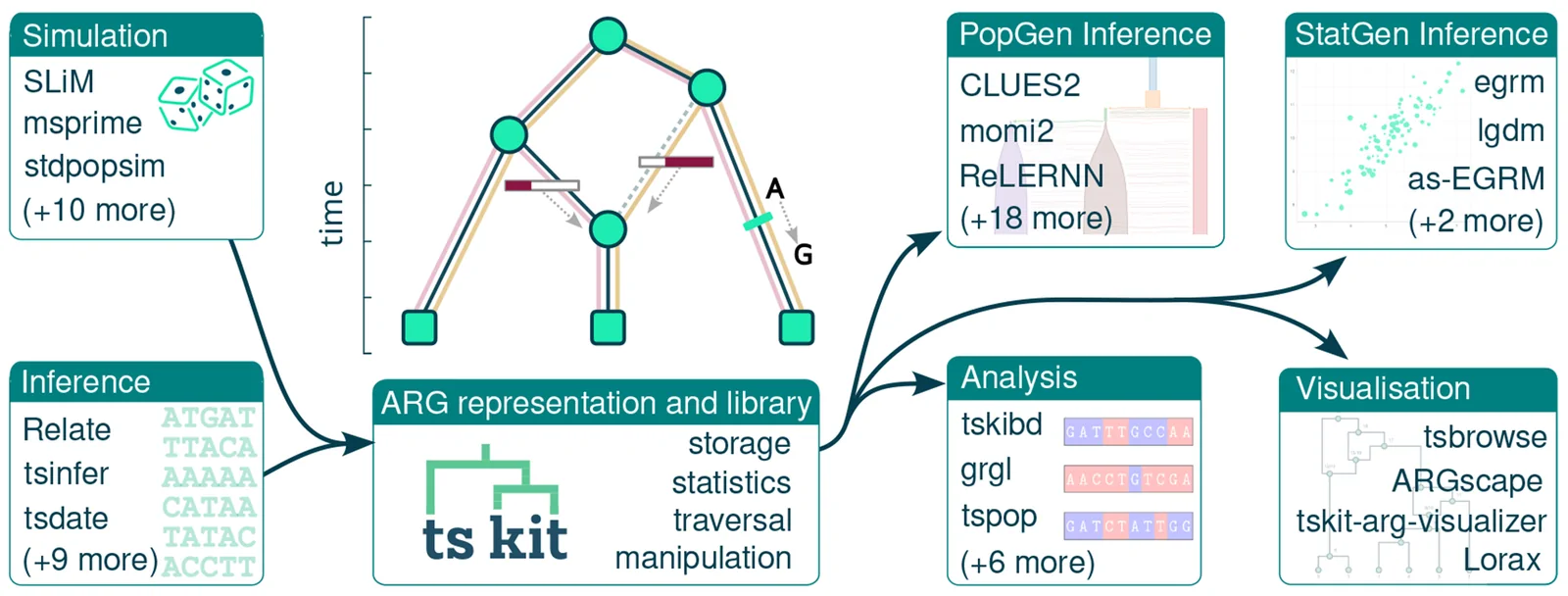
Tskit enables an interoperable ARG software ecosystem. ARGs produced by simulation or inference tools can be analysed by diverse downstream applications via tskit’s well-defined tabular data model, C library and Python/Rust/R bindings. Tools shown are representative examples from Table S1 (three per category; ordered by citation count).
