# Supplementary material for: Population-scale Ancestral Recombination Graphs with tskit 1.0
Source: ArXiv. 2026 Sep 8:arXiv:2602.09649v3. Preprint. [Version 3] (PMC13622864)
Supplement: Supplementary file 1 [file NIHPP2602.09649v3-supplement-1.pdf]

Supplementary Information

This section contains the supplementary information, which we integrate into the main document for ease of review.

Published tools using tskit

We performed a search for published software using tskit. To be included in this list, the software must be described in a publication as a named tool intended for reuse and must use tskit. This list is intentionally conservative, and does not include (for example) publications depending on bespoke analysis pipelines using tskit. For each tool in Table S1, we list: its name (with a clickable hyperlink to its source code repository); the main implementation language(s) of the tool based on GitHub summaries; the DOI (with clickable hyperlink) of the publication in which this tool was first described (or, in the case of SLiM, the first publication in which tskit-based features were described); and the year of publication and number of citations to this publication according to data from OpenAlex (`https://openalex.org`) retrieved on 2026-04-27.

Table S1: Summary of 64 published software tools using tskit. See the supplementary text for details on the columns and inclusion criteria.

Name	Language	Publication	Year	Cites
**Visualisation (4)**				
Lorax	Python, JavaScript	10.64898/2026.02.19.706861	2026	0
tsbrowse	Python	10.1093/bioinformatics/btaf393	2025	1
ARGscape	TypeScript, Python	10.48550/arxiv.2510.07255	2025	0
tskit-arg-visualizer	Python, JavaScript	10.1093/bioadv/vbaf302	2024	0
**Statistical Genetic Inference (5)**				
tslmm	Python	10.1101/2025.07.14.664631	2025	2
as-eGRM	Python, C	10.1016/j.ajhg.2025.06.016	2025	2
arg-lmm	Python	10.1016/j.xgen.2025.101072	2025	0
lgdm	Python, Matlab	10.1038/s41588-023-01487-8	2023	39
egrm	Python, C	10.1016/j.ajhg.2022.03.016	2022	47
**Analysis (9)**				
PolarBEAR	Python	10.1111/1755-0998.70105	2026	1
bio2zarr	Python	10.1093/gigascience/giaf049	2025	3
tscompare	Python	10.1093/genetics/iyaf198	2025	2
TwisstNTern	Python	10.32942/x29941	2025	0
twisst2	Python	10.1093/genetics/iyaf181	2025	0
tskibd	C++	10.1038/s41467-024-46659-0	2024	33
grgl	C++	10.1038/s43588-024-00739-9	2024	11
gfkit	C++	10.4230/lipics.wabi.2024.5	2024	0

Continued on next page

Table S1: Summary of 64 published software tools using tskit. See the supplementary text for details on the columns and inclusion criteria. (Continued)

Name	Language	Publication	Year	Cites
tspop	Python	10.1093/bioadv/vbad163	2023	14
**ARG Inference (12)**				
Moonshine.jl	Julia	10.3389/fgene.2026.1753780	2026	0
SINGER	C++, Python	10.1038/s41588-025-02317-9	2025	14
POLEGON	C++, Python	10.1073/pnas.2504461122	2025	1
sticcs	Python	10.1093/genetics/iyaf181	2025	0
Threads	C++, Python	10.1101/2024.08.31.610248	2024	25
ARGneedle	Python, C++	10.1038/s41588-023-01379-x	2023	115
espalier	Python	10.1093/sysbio/syad040	2023	15
sc2ts	Python	10.1101/2023.06.08.544212	2023	14
tsdate	Python	10.1126/science.abi8264	2022	196
ARGinfer	Python	10.1371/journal.pcbi.1009960	2022	37
Relate	C++	10.1038/s41588-019-0484-x	2019	584
tsinfer	C, Python	10.1038/s41588-019-0483-y	2019	379
**Simulation (13)**				
pyslim	Python	10.1101/2025.09.30.679676	2025	1
tstrait	Python	10.1093/bioinformatics/btae334	2024	10
slendr	R	10.24072/pcjournal.354	2023	15
cnasim	Python	10.1093/bioinformatics/btad434	2023	11
gridCoal	Python	10.1111/1755-0998.13676	2022	5
Geonomics	Python	10.1093/molbev/msab175	2021	20
necsim-rust	Rust	10.48550/arxiv.2108.05815	2021	0
stdpopsim	Python	10.7554/elife.54967	2020	248
RADinitio	Python	10.1111/1755-0998.13163	2020	56
ipcoal	Python	10.1093/bioinformatics/btaa486	2020	6
fwdpy11	C++, Python	10.1534/genetics.119.302662	2019	80
SLiM	C++, C	10.1093/molbev/msy228	2018	882
msprime	Python, C	10.1371/journal.pcbi.1004842	2016	824
**Population Genetic Inference (21)**				
gaia	R, C	10.1126/science.adp4642	2025	12
gLike	Python, C	10.1038/s41588-025-02129-x	2025	10
dolores	Python	10.1093/molbev/msaf190	2025	6
sparg	Python	10.1093/g3journal/jkaf214	2025	3

Continued on next page

Table S1: Summary of 64 published software tools using tskit. See the supplementary text for details on the columns and inclusion criteria. (Continued)

Name	Language	Publication	Year	Cites
phlash	Python	10.1038/s41588-025-02323-x	2025	2
spectre	Python	10.1093/genetics/iyaf184	2025	1
mrpast	C++, Python	10.1101/2025.10.07.680347	2025	0
spacetrees	Python	10.7554/elife.72177	2024	22
mapNN	Python	10.1111/1755-0998.14005	2024	4
gIMble	Python	10.1371/journal.pgen.1010999	2023	36
coaldecoder	C++, R	10.1073/pnas.2208116120	2023	31
disperseNN2	Python	10.1186/s12859-023-05522-7	2023	12
dinf	Python	10.1101/2023.04.27.538386	2023	8
GADMA	Python	10.1093/gigascience/giad059	2022	24
SCAR	Python	10.1371/journal.pcbi.1010422	2022	12
dnadna	Python	10.1093/bioinformatics/btac765	2022	9
Colate	C++	10.1093/molbev/msab174	2021	72
DISMaL	Python	10.1016/j.tpb.2021.03.001	2021	7
ReLERNN	Python	10.1093/molbev/msaa038	2020	182
CLUES2	Python	10.1371/journal.pgen.1008384	2019	179
momi2	Python	10.1080/01621459.2019.1635482	2019	179
